# Heart rate and activity patterns of chinook salmon (*Oncorhynchus tshawytscha*) under steady and unsteady flow conditions

**DOI:** 10.1242/jeb.251222

**Published:** 2026-03-30

**Authors:** Wisdom E. K. Agbeti, Leonardo Magnoni, Suzy Black, Arjan P. Palstra

**Affiliations:** ^1^ Animal Breeding and Genomics, Wageningen University & Research, 6708 PB Wageningen, The Netherlands; ^2^ Plant & Food Research, Port Nelson, Nelson 7010, New Zealand

**Keywords:** Swimming physiology, Offshore aquaculture, Sensor co-implantation, Fish behaviour, Exercise

## Abstract

This study investigated how unsteady flow conditions influence the swimming physiology and energetic performance of Chinook salmon using co-implanted heart rate (HR) and acceleration sensors. Fish were monitored for HR, acceleration and overall dynamic body acceleration (ODBA) in two experimental settings: (1) controlled swimming at increasing speeds (0.15–0.90 m s^−1^) in a swim tunnel under steady and unsteady flow, and (2) free-swimming sentinel fish in tanks under steady and subsequent unsteady flow for 2 weeks each. In experiment 1, HR remained consistently high (81–84 beats min^−1^) across all speeds under both flow conditions, suggesting limited capacity to further elevate cardiac output. *Ṁ*_O_2__ increased from 213±10 to 307±16 and from 225±12 to 330±17 mg kg^−1^ h^−1^ under steady and unsteady flow, respectively. Acceleration and ODBA increased linearly with speed and were positively correlated under both flow conditions. In experiment 2, circadian patterns were evident in HR, acceleration and ODBA of the free-swimming fish. Fish exhibited higher daytime and night-time HR and acceleration under unsteady flow compared with steady flow conditions, whereas ODBA remained similar. Regression models based on swim tunnel data accurately predicted acceleration and ODBA in free-swimming fish, indicating consistent relationships between swimming speed and acceleration dynamics. The higher HR and acceleration of free-swimming fish under unsteady conditions indicated a 3–5% increased energetic investment. Overall, this study provides insight into how dynamic flow environments shape the physiological responses of Chinook salmon, informing predictions of fish performance in offshore aquaculture systems.

## INTRODUCTION

In natural aquatic environments, fish often experience unsteady or turbulent flow conditions that vary across temporal and spatial scales. Such flows are common in rivers, coastal habitats and open ocean environments where currents interact with complex topography, tides and waves (Cote and Webb, 2015). The magnitude and frequency of these fluctuations can range from small-scale eddies (<10 cm, <1 Hz) to large-scale oscillations driven by tides and waves (0.01–0.1 Hz), creating highly complex hydrodynamic environments (Denny, 2006). These unsteady flow regimes influence fish swimming kinematics, station-holding ability and energetic efficiency (Liao, 2007; Roche et al., 2014; Schakmann et al., 2020; Taguchi and Liao, 2011). Understanding how fish adjust cardiovascular and locomotor responses to such unsteady conditions provides key insights into their physiological plasticity and adaptive strategies.

The use of miniaturized sensors with improved battery life is enriching the research field of the swimming physiology of fish. They have enabled the real-time and long-term monitoring of fish behaviour and physiology of farmed finfish species under various conditions, such as heat waves (Gamperl et al., 2021), winter conditions (Sandrelli et al., 2025), unsteady flows (Agbeti et al., 2024) and aquaculture practices such as harvesting (Brijs et al., 2018), crowding and delousing events (Føre et al., 2018). Acoustic transmitters (which transmit data directly) and data loggers (which store data for later retrieval) are two widely applied tools that provide valuable data on temperature, depth, acceleration patterns and heart rate (HR) of fish (Bjarnason et al., 2019; Cooke et al., 2012; Kolarevic et al., 2016). HR is a critical indicator of metabolic activity and stress of fish, whereas acceleration data offer insights into swimming activity and energy expenditure of fish. Sensor implants have been successfully used in salmonid species, particularly Atlantic salmon (*Salmo salar*; Agbeti et al., 2024, 2025; Føre et al., 2021; Warren-Myers et al., 2023; Zrini and Gamperl, 2021), and non-salmonid species such as yellowtail kingfish *Seriola lalandi* (Palstra et al., 2024) and European sea bass *Dicentrarchus labrax* (Wright et al., 2014), to study exercise physiology and energy metabolism. These studies provide species-specific insights into the impact of environmental changes, and knowledge for welfare improvement and precision farming.

Chinook salmon (*Oncorhynchus tshawytscha*) is the only finfish species farmed in ocean-based production systems in New Zealand, representing a high commercial value. The mariculture of Chinook salmon primarily takes place in sheltered coastal areas, such as the Marlborough Sound, where water flow speeds range between 0.03 and 0.22 m s^−1^ (Hudson Associates, 2019). Recently, there has been increasing interest in moving mariculture operations from sheltered regions to more exposed offshore sites to facilitate industry expansion, also considering the increasing occurrence of heat waves in coastal areas with negative effects on fish production (Cheung et al., 2021; Smith et al., 2023). This shift is also driven by regulatory challenges related to environmental concerns in current production areas, competition for coastal space and the New Zealand government's commitment to a sixfold increase in aquaculture productivity by 2035 through offshore mariculture (Ministry for Primary Industries, 2024). Consequently, industry players have begun planning grow-out sites in these exposed offshore environments (RNZ, 2024). However, water flow speeds in exposed sites can reach up to 1.5 m s^−1^ (Hudson Associates, 2019; Prescott et al., 2023), potentially influencing fish physiology, behaviour and growth performance. Understanding how Chinook salmon respond to these novel flow regimes and the associated energetic costs is critical, particularly under unsteady flow conditions that typify offshore aquaculture systems. Here, we focused on unidirectional unsteady flows, where water speed changes over time.

Unlike Atlantic salmon, Chinook salmon remain relatively understudied. Atlantic salmon has gained research attention on its swimming performance and the exercise effects on growth (Hvas et al., 2021a,b; Palstra et al., 2020; Remen et al., 2016; Timmerhaus et al., 2021). Several studies on Atlantic salmon also investigated the effect of unsteady flow conditions on *Ṁ*_O_2__ and growth (Agbeti et al., 2024; Barbier et al., 2024). Previously, we studied the overall dynamic body acceleration (ODBA; summation of tri-dimensional body acceleration), as measured by acoustic transmitter tags, of Atlantic salmon (∼300 g) in relation to their oxygen consumption (*Ṁ*_O_2__) under steady and unsteady flow conditions (Agbeti et al., 2024). Subsequently, we linked heart rate (HR) and external acceleration dynamics of Atlantic salmon to *Ṁ*_O_2__ during swimming and during a stress challenge test as recorded by implantable HR and acceleration loggers (Agbeti et al., 2025). Although some studies have investigated the swimming performance and exercise effects in Chinook salmon (Gallaugher et al., 2001; Prescott et al., 2023, 2024; Thorarensen et al., 1993), these studies were implemented under steady flow conditions and did not examine the relations between HR, acceleration and *Ṁ*_O_2__, and how these parameters may be different under unsteady flow conditions. This information is highly relevant for salmon farming in offshore aquaculture systems.

The first objective of the study, therefore, was to evaluate changes in HR and activity patterns (ODBA and acceleration) in co-implanted individuals in a swim tunnel under different swimming conditions (unsteady versus steady, under incremental flow speeds) while monitoring *Ṁ*_O_2__. The second objective was to evaluate how HR and activity patterns in co-implanted free-swimming fish – used as sentinel individuals to represent group responses within duplicate flow-through tanks – varied under steady or unsteady flow conditions. Additionally, HR and activity patterns were compared between salmon swimming individually in the swim tunnels or as a free-swimming group in the tanks. As such, we intended to answer the research questions: how do HR and acceleration patterns of Chinook salmon vary when swimming (1) at increasing speeds in a swim tunnel and (2) at a constant speed in a tank, either under steady or unsteady flow conditions? We hypothesized that Chinook salmon swimming in unsteady flow at increasing speeds would exhibit higher *Ṁ*_O_2__ compared with fish swimming under steady flow conditions. Higher *Ṁ*_O_2__ is expected to match the findings for Atlantic salmon (Agbeti et al., 2024). Additionally, we hypothesized that ODBA and acceleration values, as derived from the sensors that were co-implanted, would correlate positively with each other as both parameters are based on mathematical norms that can be used to quantify fish movement. Lastly, we hypothesized that HR, acceleration and ODBA values of Chinook salmon would be higher under unsteady flow compared with steady flow conditions in free-swimming fish because of the need to maintain position and stability, which might lead to increased energy use.

## MATERIALS AND METHODS

### Experimental fish and husbandry

All experiments were conducted between August and November 2023 at the Plant & Food Research's Maitai Finfish Facility in Nelson, Aotearoa/New Zealand. Prior to experimentation, all female Chinook salmon smolts [initial body mass (BM)=80±10 g (mean±s.e.m.); standard length (SL)=20 cm; age=13 months] were sourced from Salmon Smolt NZ (Kaiapoi, North Canterbury, New Zealand) and transported to the facility. Upon arrival, fish were held in two 13 m^3^ circular tanks containing 5 ppt brackish water filtered through activated carbon pleated cartridges. Salinity was gradually increased to 34 ppt over 3–4 h, followed by a 14 day acclimation period. Tanks operated as a flow-through system with filtered, UV-treated and temperature-regulated seawater. Fish were hand fed Skretting Orient Premium pellets (2% BM day^−1^) three times daily, with pellet size adjusted to growth. Water quality (NH_4_^+^, NO_3_^−^, NO_2_^−^, pH) was monitored biweekly, and dissolved oxygen (>95% saturation) and temperature (13±0.1°C) were checked daily. Water renewal was set at 500 l min^−1^. After acclimation, fish were reared for ∼8 weeks until reaching an average body mass of 460±17 g, when individual and group trials began.

Experimental protocols complied with the current laws of New Zealand and Plant and Food Research's finfish culture and husbandry practices, and followed specific requirements described in the animal use application approved by the Nelson Marlborough Institute of Technology Animal Ethics Committee (AEC2023-PFR-02).

### Swim tunnel, flow calibration and respirometry

A Brett-type swim flume (185 l total volume; test section: 88×25×25 cm) was used to test individual fish. The flume was submerged in seawater (34 ppt) and maintained at 13±0.1°C via a titanium cooling coil connected to a chilled water system (4–6°C). Flow was generated by an electric motor (WEG W22 High Eff., 4 kW, Tauriko Tauranga, New Zealand) driving a propeller, with a flow straightener (0.5 cm in diameter) ensuring laminar flow and a metal grid (0.6×0.6 cm; width×height) preventing fish escape. Flow speeds were measured and calibrated using a Höntzsch HFA flow meter (e29/213; Höntzsch GmbH & Co., Waiblingen, Germany). To simulate unsteady flow conditions, flow speed followed a sinusoidal pattern (1/12 Hz frequency, 12 s wave period, ±0.1 m s^−1^ amplitude), controlled by a Proportional-Integral-Derivative (PID) controller. These settings were chosen to reflect the short-term velocity fluctuations experienced by Chinook salmon in nearshore and sheltered mariculture sites (Newcombe et al., 2019), ensuring ecologically relevant swimming challenges while remaining within the physiological capacity of the fish. Oxygen levels were monitored every two seconds using a Firesting Optical Oxygen Meter (Pyro-science, Aachen, Germany). Oxygen consumption rate (*Ṁ*_O_2__; in mg kg^−1^ h^−1^) and cost of transport (COT; in mg kg^−1^ km^−1^) were calculated with the following equations:
(1)

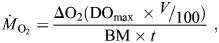
where ΔO_2_ is the percentage decline in oxygen concentration, DO_max_ (mg l^−1^) is the maximum dissolved oxygen in seawater, *V* (30 l) is the swim tunnel volume, BM (kg) is fish body mass, and *t* is time in hours.
(2)

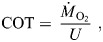
where *Ṁ*_O_2__ is the consumed oxygen (mg kg^−1^ h^−1^) and *U* is the speed in kilometres per hour (km h^−1^).

### Sensor tag implantation

Experimental fish (Table 1) underwent surgery to implant sensor tags in three configurations: (1) an acoustic accelerometer transmitter (‘transmitter’; Thelma Biotel, model A-LP7; 7.3×17 mm; 1.9 g; 139 dB; 25 Hz; 71 kHz; 30–50 s interval), (2) a data logger (‘logger’; Star-Oddi DST milli-HRT ACT; 13×39 mm; 12 g), or (3) both devices (‘co-implant’). A fourth group received sham surgery without implantation, while a fifth group served as non-surgical controls.

**Table 1.** Experimental groups of post-smolt Chinook salmon used in the swim tunnel and tank-based free-swimming experiments under two flow conditions (steady versus unsteady flow)

Groups	Sample size (*n*)	
	Steady flow	Unsteady flow
Control fish	12	12
Sham	4	4
Fish with only data logger	4	4
Fish with only transmitter	4	4
Fish with co-implant (swim tunnel trial)	8	8
Fish with co-implant (tank-based trial)	10	

Surgical methods followed Agbeti et al. (2024) and Palstra et al. (2024). Fish were randomly netted and anesthetized with 150 mg l^−1^ MS222 buffered with 300 mg l^−1^ NaHCO₃. After measuring mass and length, fish were placed ventral side up for surgery. Continuous gill aeration was maintained with 75 mg l^−1^ MS222 and 150 mg l^−1^ NaHCO₃. Instruments were sterilized in 70% ethanol. Lidocaine (100 µl) was applied locally for pain relief, followed by a ∼2 cm ventral incision. Devices were inserted into the intraperitoneal cavity of fish; loggers were positioned near the heart and secured with two knotted single sutures. An Ethicon Perma-hand silk black braided 2-0 (3.0 metric) suture and FSL 30 mm 3/8 c reverse cutting needle were used to close the incision with simple interrupted sutures. Visible implant elastomer (Northwest Marine Technology Inc., Anacortes, WA, USA) was injected near the adipose eyelid. Post-surgery, fish received intramuscular Ketoprofen (2 mg kg^−1^) for analgesia. Each surgery lasted 15–20 min. Fish were first recovered in oxygenated water, then treated for 1 h in a 10 ppm Halamid bath. They were subsequently housed in 1500 litre tanks for a minimum 10 days of recovery before experiments began.

### Data logger programming and data processing

Before implantation, data loggers were configured using Star-Oddi's COM-BOX interface and Mercury software (v.6.53). Parameters such as measurement start time, sampling frequency, and sampling intervals were programmed. After the experiment, logger data were downloaded via the same interface. Acceleration was recorded as average external acceleration (m***g***) and calculated as the vectorial sum of dynamic body acceleration, removing static acceleration due to gravity. HR, in beats per minute (beats min^−1^), was derived from ECG signals recorded at 200 Hz every 10 min for 7.5 s. This sampling duration followed the manufacturer's recommendations and has been validated for accurate HR estimation in salmonids (Brijs et al., 2018; Zrini and Gamperl, 2021). ECG waveforms were stored on the logger for post-analysis to ensure accuracy. Signal quality was assessed using the logger's quality index (QI): QI_0_ equals excellent, QI_1_ to QI_2_ equals decreasing quality, and QI_3_ equals no R–R interval detected. HR data with QI_3_ were excluded, and HR values with QI_1_ or QI_2_ were manually reviewed and corrected if necessary. For the respirometry experiment, three HR and acceleration measurements were recorded per swimming speed; pre-trial values in holding tanks served as baselines. In the tank-based experiment, variance of acceleration (VAR, in m***g***^2^) was also measured, in addition to HR and acceleration. An acoustic receiver (Thelma Biotel, model TBR 700; diameter: 75 mm; diameter: 75 mm; length: 230 mm) was placed in the swim tunnel's water bath, near the metal grid, to capture data from implanted transmitters. The transmitter's onboard algorithm computed overall dynamic body acceleration (ODBA) by summing three-dimensional acceleration (**a***_x_*=surge, **a***_y_*=sway, **a***_z_*=heave) and subtracting static components. Transmitted data were decoded according to the manufacturer's instructions.

### Swimming exercise protocol

The swim tunnel was filled with seawater (34 ppt) and maintained at 13±1°C. Water circulation was ensured by a 40 l min^−1^ Eheim pump. On alternate days, one fish was randomly selected from the 1500 litre holding tanks, sedated in 10 ppm Aqui-S mixed with aerated water, and transferred to the tunnel. This alternating schedule minimized tank-specific sampling effects and ensured consistent pre-test fasting.

Each fish was placed in the swim section, and the lid secured. Inlet and outlet valves were opened, and flow was set at 0.15 m s^−1^ for a 2 h recovery and acclimation period. Fish from each group (Table 1) swam individually. The critical swimming speed (*U*_crit_) test started at 0.30 m s^−1^ and increased by 0.15 m s^−1^ every 30 min until the tunnel's maximum speed [0.9±0.1 m s^−1^ ≈3 body lengths s^−1^ (BL s^−1^)] was reached. Each measurement cycle consisted of a 5 min acclimation period to the set swimming speed, followed by a 15 min measurement phase and a 10 min flush period. Fatigue was defined as the fish remaining on the rear grid for >20 s without resuming swimming. After fatigue, fish were euthanized with an overdose of Aqui-S (≥100 ppm) in accordance with approved animal ethics protocols. Body mass (BM) and standard length (SL) were recorded to calculate Fulton's condition factor (*K*), and implanted devices retrieved when applicable. *U*_crit_ was calculated as follows:
(3)

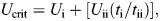
where *U*_crit_ is the critical swimming speed in m s^−1^ (absolute *U*_crit_), *U*_i_ is the highest velocity completed before exhaustion in m s^−1^, *U*_ii_ is the prescribed velocity increment in m s^−1^, *t*_i_ is the time to fatigue at final velocity level in minutes, and *t*_ii_ is the prescribed time interval (=30 min).

To determine optimum swimming speed (*U*_opt_), cost of transport (COT) was plotted against swimming speed, and a second-degree polynomial was fitted. *U*_opt_ was identified at the lowest COT point, where the first derivative=0 (Palstra and van den Thillart, 2010; Palstra et al., 2008). Oxygen consumption (*Ṁ*_O_2__) was recorded every 15 min at each speed. Background respiration (without fish) was measured post-trial using the same protocol and subtracted from *Ṁ*_O_2__ values. Correction for solid blocking was negligible, as fish occupied <10% of the swim section's cross-sectional area (Bell and Terhune, 1970).

### Tank-based experimental setup and fish husbandry

**Fig. 1.**
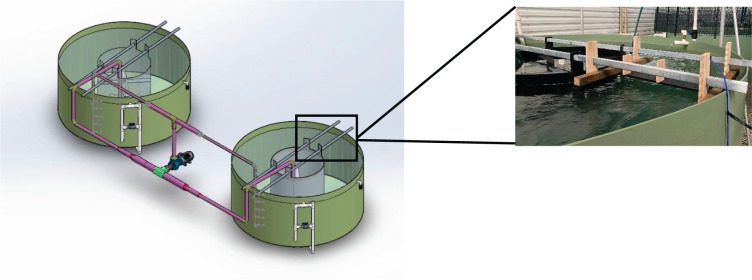
**Schematic of the experimental tank system used to monitor heart rate, acceleration and overall dynamic body acceleration in sentinel salmon within a larger group of free-swimming fish.** Tanks were operated under both steady (0.50 m s^−1^) and unsteady (0.50±0.05 m s^−1^) flow conditions. Filtered seawater entered each tank via an upper inlet valve (white pipeline) and exited through a central bottom outlet. A closed-loop system (purple pipelines), driven by a centrifugal pump, ensured identical flow profiles between the two tanks.

**Fig. 2.**
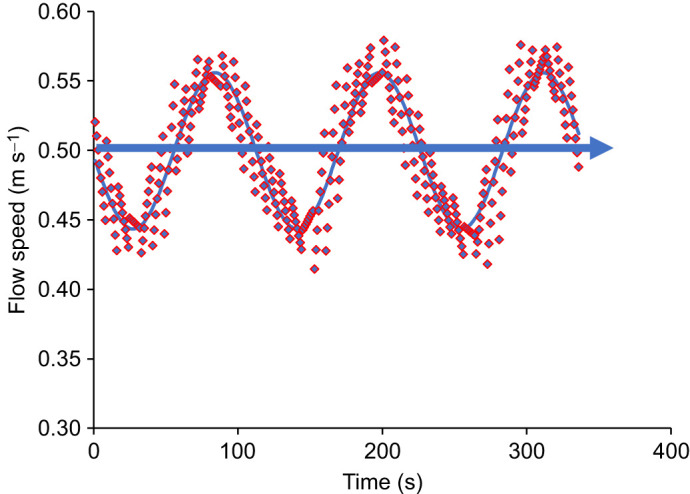
**Determination of the optimal swimming speed (*U*_opt_) for Chinook salmon (*Oncorhynchus tshawytscha*), indicated by the blue arrow at 0.50 m s^−1^, which was used to set flow speeds in the trial.** In the unsteady flow treatment, water velocity was modulated at a frequency of 1/100 Hz (period=100 s) with a wave amplitude of ±0.05 m s^−1^. Blue dots represent flow values measured in the tanks, and a sinusoidal function was fitted to these data points.

The experiment was conducted in two circular tanks (diameter: 3.5 m; wall height: 1.6 m; water depth: 1.2 m) made of UV-stabilized, food-grade polyethylene and connected to a flow-through system (Fig. 1). Filtered seawater (34 ppt) was supplied via an upper inlet valve and drained from the bottom centre outlet. Each tank contained an inner ring (1.6 m diameter) to aid in generating laminar flow. A central cross-pole and two perpendicular stabilizing poles anchored the ring. Perforations (3 cm diameter, 6 cm above the tank floor) at the base of the ring allowed water exchange while mesh prevented fish escape. An independent flow loop connected both tanks, drawing water from the inner ring centers and circulating it through a centrifugal pump (WEG W22, 11 kW, 1000 l min^−1^, Tauriko Tauranga, New Zealand). The loop outflow returned into tanks via three vertical pipes with unidirectional outlets, placed between the ring and tank wall. The system was controlled by a Schneider Variable Speed Drive and a Siemens S7-1212 PLC using PID feedback control. This setup maintained either a constant or sinusoidal flow pattern to simulate tidal conditions. Flow speeds were calibrated using a vane wheel flow sensor (Höntzsch GmbH & Co. KG, Waiblingen, Germany), measured at three positions (90, 180 and 270 deg), depths and lengths. Maximum steady speed was 0.85 m s^−1^. The unsteady flow was set at 0.50±0.05 m s^−1^ with a wave period of 100 s (1/100 Hz), the longest achievable in the setup (Fig. 2). Two groups of 230 Chinook salmon (BM=460±17 g; SL=30±1 cm) and five co-implanted fish per tank were lightly sedated with 10 ppm Aqui-S during handling and transfer, then, randomly assigned to each tank (total=235 fish per tank). Fish were acclimated for 7 days at a flow speed of 0.15 m s^−1^ before trials began. Flow was then gradually increased over 1 h to 0.50 m s^−1^ and maintained for 2 weeks. Subsequently, unsteady flow (0.50±0.05 m s^−1^) was applied for another 2 weeks, followed by a final recovery phase at 0.15 m s^−1^ for 1 week. The experiment ran from September to November 2023, during which natural photoperiod ranged from 11.5 to 14.5 h of daylight. Fish were fed twice daily (09:00 h and 16:00 h) at 2% BM day^−1^. Sensor data were categorized as daytime (06:00–17:00 h) and night-time (18:00–05:00 h). Tanks were inspected daily, with dead fish removed and biometric data collected at the start and end of the study.

### Data analysis

Statistical analyses were performed using R software (version 4.3.0; https://www.r-project.org/). The lme4 and lmerTest packages (https://CRAN.R-project.org/package=lme4; https://CRAN.R-project.org/package=lmerTest) were utilized for fitting statistical models. Prior to analysis, data were checked for normality and homoscedasticity to ensure compliance with model assumptions. Differences in BM, SL and *K* among groups were evaluated using a one-way ANOVA, with *post hoc* comparisons conducted using Tukey's test. If the assumptions for parametric tests were violated, data were either log-transformed or analysed using non-parametric Kruskal–Wallis test, followed by Dunn's test with Bonferroni adjustment for multiple comparisons.

To investigate the relationships between *Ṁ*_O_2__, HR, acceleration, VAR and ODBA with swimming speed, as well as to evaluate the effects of logger implantation in the swim tunnel, a linear mixed model (LMM) was applied. The model was fitted using restricted maximum likelihood (REML) and had the following general structure:
(4)

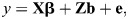
where: *y* represents the response variable (*Ṁ*_O_2__, HR, acceleration, VAR or ODBA); **X** is the design matrix for fixed effects, with vector **β** containing coefficients accounting for swimming speed and logger presence; **Z** is the design matrix for random effects, with vector **b** representing inherent biological variability among individual fish; **e** denotes the vector of residual errors.

LMM was also applied to the dataset from the free-swimming sentinel fish in tanks to account for its repeated-measures structure. The model incorporated Treatment (steady versus unsteady flow) and Period of day (day versus night) as fixed effects, and LoggerID as a random effect to account for repeated HR measurements from the same individuals: HR∼Treatment+Period_of_Day+(1 | LoggerID).

The model assumes that fixed effects, random effects, and residuals are independent, and that residuals are normally distributed. Potential interaction effects between explanatory variables were tested and included in the model if found to be significant. Two-tailed Pearson correlation test was used to establish the relationship between ODBA and acceleration values of co-implanted fish during swim tunnel test. Statistical significance was determined at *P*<0.05, and results are presented as means±s.e.m unless otherwise specified.

## RESULTS

### Biometric parameters of fish used in the respirometry study

Fish in the swim tunnel study (Table S1) had BM ranging from 417 to 562 g and SL of 30 to 32 cm, with no significant differences between control, sham, transmitter, logger, or co-implanted groups under steady or unsteady flow (Kruskal–Wallis, *P*>0.05). Similarly, no differences in BM, SL or *K* (mean 1.56–1.72) were found when comparing the same groups across flow conditions (Wilcoxon test, *P*>0.05).

### Respirometry

#### Oxygen consumption

**Fig. 3.**
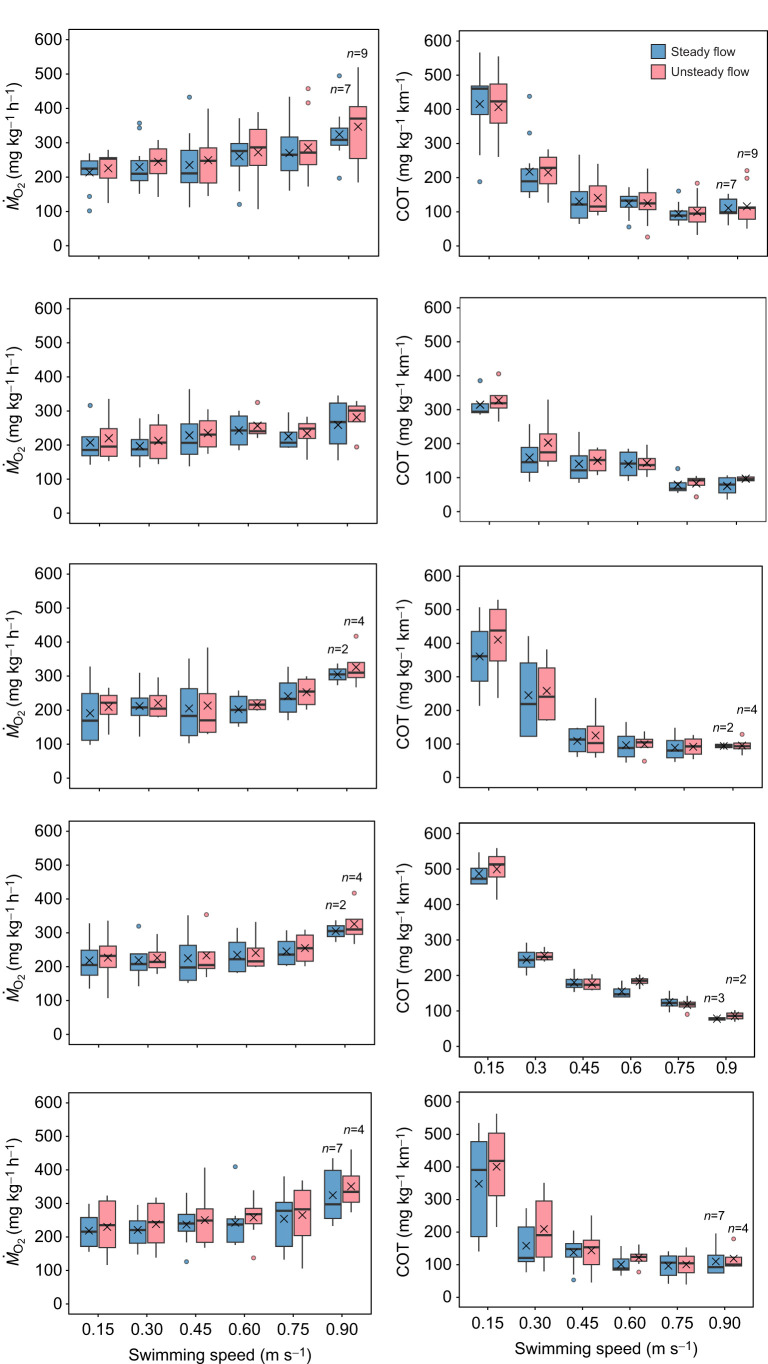
**Oxygen consumption (*Ṁ*_O_2__) and cost of transport (COT) of different Chinook salmon groups across swimming speeds under both steady and unsteady flow conditions.** (A–E) *Ṁ*_O_2__ versus swimming speed for control (*n*=12), sham (*n*=4), transmitter (*n*=4), logger (*n*=4) and co-implanted (*n*=8) groups, respectively. (F–J) Corresponding COT values for the same groups. Box plots show the median (horizontal line), interquartile range (box) and whiskers (minimum–maximum range). Crosses represent mean values and dots represent outliers. No significant differences in *Ṁ*_O_2__ or COT were observed between groups or flow conditions (LMM, *P*>0.05).

*Ṁ*_O_2__ increased linearly with swimming speed across all groups under both steady and unsteady flow (Fig. 3A and Fig. S1). Compared with 0.30 m s^−1^, *Ṁ*_O_2__ was significantly higher at 0.60, 0.75 and 0.90 m s^−1^, but not at 0.45 m s^−1^ (LMM, *P*<0.05). Implants had no significant effect on *Ṁ*_O_2__ compared with controls across all speeds and flow conditions (LMM, *P*<0.05). Although *Ṁ*_O_2__ was 5–15% higher under unsteady flow, the difference was not significant, but a weak trend existed (LMM, *P*=0.06).

#### Cost of transport, optimal swimming speed and critical swimming speed

COT values did not differ significantly between experimental groups under either steady or unsteady flow (LMM, *P*>0.05; Fig. 3B). Polynomial COT curves showed a typical U-shape, with COT decreasing significantly with increasing speed until reaching a minimum, after which it rose again (*P*<0.05). This pattern was consistent across flow conditions. Under steady flow, COT ranged from 516 to 75 mg O_2_ kg^−1^ km^−1^ (0.15–0.9 m s^−1^), and under unsteady flow, from 614 to 79 mg O_2_ kg^−1^ km^−1^. Minimum COT (COT_min_) under steady flow ranged from 68 to 103 mg O_2_ kg^−1^ km^−1^ and from 68 to 108 mg O_2_ kg^−1^ km^−1^ under unsteady flow, with no significant differences among groups (Kruskal–Wallis, *P*>0.05). COT_min_ values also did not differ significantly between flow conditions (Wilcoxon test, *P*>0.05).

The *U*_opt_ for fish from each group under steady flow conditions ranged from 0.52±0.06 to 0.62±0.02 m s^−1^ and, under unsteady flow conditions, from 0.46±0.05 to 0.66±0.04 m s^−1^ (Table S2). There were no significant differences in *U*_opt_ values between groups within each flow condition (*P*>0.05), nor were there significant differences between groups under different flow conditions (Wilcoxon rank sum test, *P*>0.05).

*U*_crit_ values of fish that fatigued representing the experimental groups ranged from 0.83±0.04 to 0.88±0.02 m s^−1^ under steady flow and from 0.82±0.03 to 0.90±0.03 m s^−1^ under unsteady flow, Within the co-implanted group, 75% of the fish swimming under the steady flow conditions did not fatigue during trials whereas under unsteady flow condition, half of the fish completed the swim test without fatiguing at the maximum tunnel speed of 0.9±0.1 m s^−1^.

#### Heart rate and acceleration in the swim tunnel

Fish implanted with a single logger (*n*=4) showed no significant differences in heart rate (HR) response to increasing swimming speeds as compared with co-implanted fish (*n*=8) under steady or unsteady flow (LMM, *P*>0.05). Similarly, acceleration did not differ significantly between groups or flow conditions (LMM, *P*>0.05), allowing HR and acceleration data to be pooled for further analysis.

**Fig. 4.**
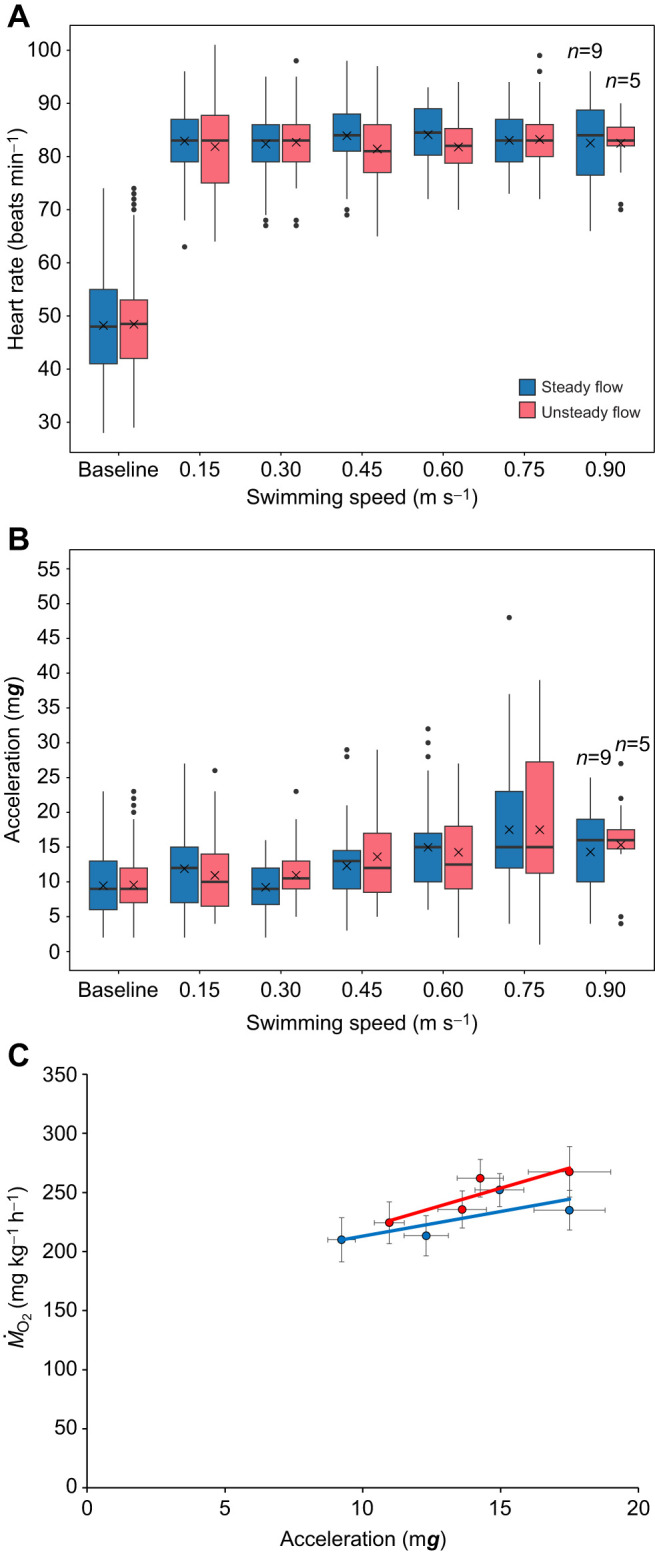
**Heart rate (HR) and acceleration of Chinook salmon in relation to swimming speed under steady and unsteady flow conditions.** (A) HR versus swimming speed, which remains stable regardless of swimming speed increments. (B) Acceleration versus swimming speed. (C) Regression of oxygen consumption (*Ṁ*_O_2__) versus acceleration shown as means±s.e.m. Box plots show the median (horizontal line), interquartile range (box) and whiskers (minimum–maximum range). Crosses represent mean values, and dots represent outliers. *N* values in A and B reflect the number of fish (out of 12) that did not fatigue at the highest speed tested. Baseline values were measured in fish swimming freely in holding tanks. From 0.30 to 0.75 m s^−1^, mean acceleration correlates positively with *Ṁ*_O_2__ under both steady (*R*^2^=0.56) and unsteady (*R*^2^=0.79) flow conditions.

Under steady flow, HR of fish (*n*=12) remained stable between 82±1 and 84±1 beats min^−1^ from 0.30 to 0.90 m s^−1^, showing no significant increase from 0.15 m s^−1^ (LMM, *P*>0.05; Fig. 4A). HR under unsteady flow was similarly stable (81±1 to 83±1 beats min^−1^). Baseline HR recorded in holding tanks (48±1 beats min^−1^, *n*=24) was significantly lower than swim tunnel values under both flow conditions (LMM, *P*<0.05). Baseline acceleration in holding tanks (9.51±0.20 m***g***) was similar to acceleration at 0.30 m s^−1^ (LMM, *P*>0.05) but higher than at 0.15 m s^–1^ (LMM, *P*=0.03) in the swim tunnel, regardless of the flow condition (Fig. 4B). Mean acceleration correlated positively with swimming speed between 0.30 and 0.75 m s^−1^ under both steady (*R*^2^=0.99; *y*=18.31*x*+3.88) and unsteady (*R*^2^=0.95; *y*=13.52*x*+6.99) flow conditions, with no significant differences (LMM, *P*>0.05). At 0.90 m s^−1^, mean acceleration decreased to statistically similar values under steady (14.30±0.88 m***g***, *n*=9) and unsteady (15.31±1.24 m***g***, *n*=5) flow conditions. Mean acceleration also correlated with mean *Ṁ*_O_2__ under steady (*R*^2^=0.56; *y*=4.17*x*+171.34) and unsteady (*R*^2^=0.79; *y*=6.85*x*+150.98) conditions (Fig. 4C).

#### Overall dynamic body acceleration (ODBA) during respirometry

ODBA values of fish with transmitters did not differ significantly between steady (*n*=4) and unsteady (*n*=4) flow across swimming speeds (LMM, *P*>0.05), and also not when compared with co-implanted fish (*n*=8) under both flow conditions (LMM, *P*>0.05). Thus, data were pooled for analysis by flow condition.

**Fig. 5.**
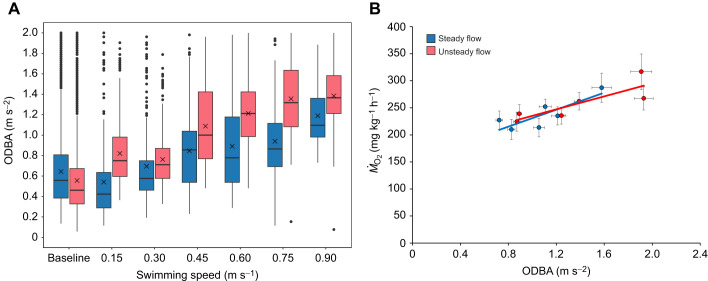
**Relationship between overall dynamic body acceleration (ODBA) and swimming speed, and its correlation with oxygen consumption (*Ṁ*_O_2__) in Chinook salmon under steady and unsteady flow in a swim tunnel.** (A) ODBA versus swimming speed (*n*=12). (B) *Ṁ*_O_2__ versus ODBA (*n*=12) shown as means±s.e.m. Box plots show the median (horizontal line), interquartile range (box), and whiskers (minimum–maximum range). Crosses represent mean values and dots represent outliers. Baseline values were recorded in holding tanks. No significant difference in ODBA was found between flow conditions (LMM, *P*>0.05). ODBA correlates positively with *Ṁ*_O_2__ under steady (*R*^2^=0.69) and unsteady (*R*^2^=0.71) flow.

Under steady flow (*n*=12), ODBA values of fish (Fig. 5A) increased linearly with swimming speed from 0.3 to 0.90 m s^−1^ (0.72±0.03 to 1.58±0.1 m s^−2^; *R*^2^=0.94; *y*=0.16*x*+0.54). Similarly, under unsteady flow (*n*=12), ODBA also increased linearly with swimming speed (0.89±0.02 to 1.91±0.1 m s^−2^; *R*^2^=0.92; *y*=0.24*x*+0.53). Higher mean ODBA was observed under unsteady flow at speeds from 0.45 to 0.90 m s^−1^ compared with steady flow. However, no significant difference in mean ODBA was found between steady and unsteady flow conditions after accounting for individual variability (LMM, *P*>0.05). ODBA correlated positively with *Ṁ*_O_2__ under both steady (*R*^2^=0.69; *y*=78.83*x*+151.99) and unsteady (*R*^2^=0.71; *y*=59.94*x*+175.28) conditions (Fig. 5B).

**Fig. 6.**
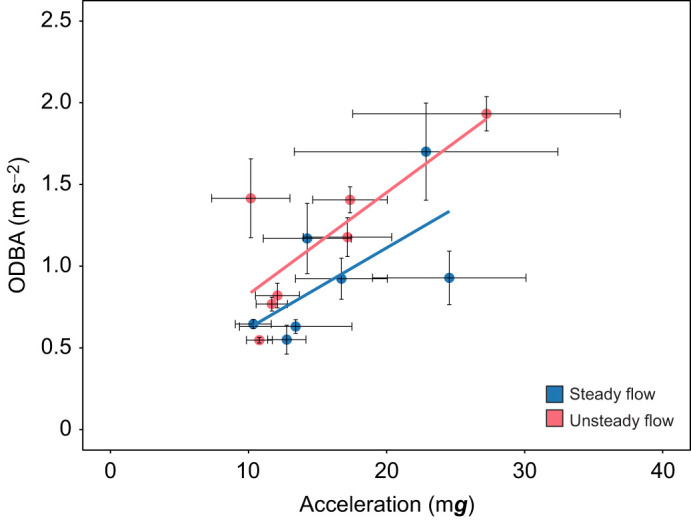
**Pearson correlation between overall dynamic body acceleration (ODBA) and external acceleration values obtained from Chinook salmon, co-implanted with an acoustic transmitter and a data logger, respectively.** Mean±s.e.m. values are shown for fish swimming under steady (blue) and unsteady (red) flow conditions from 0.15 and 0.90 m s^−1^. Mean ODBA and acceleration values were moderately and significantly correlated under both steady (*n*=12, *r*=0.43, *P*=0.002; Pearson correlation) and unsteady (*n*=12, *r*=0.45, *P*=0.002; Pearson correlation) flow conditions.

The correlation between ODBA and acceleration values of the fish that were co-implanted with acoustic transmitter and data logger respectively is shown in Fig. 6. Under unsteady flow, ODBA values showed a moderate and positive correlation with acceleration values (*r*=0.45) when swimming between 0.15 and 0.90 m s^−1^. This correlation was statistically significant (95% CI [0.17, 0.66], *t*=3.25, d.f.=42, *P*=0.002). Within the same speed range, a similar moderate and positively significant correlation was found between ODBA and acceleration values under steady flow (*r*=0.43, 95% CI [0.17, 0.63], *t*=3.31, d.f.=49, *P*=0.002).

#### Heart rate and acceleration dynamics in free swimming fish in tanks

**Fig. 7.**
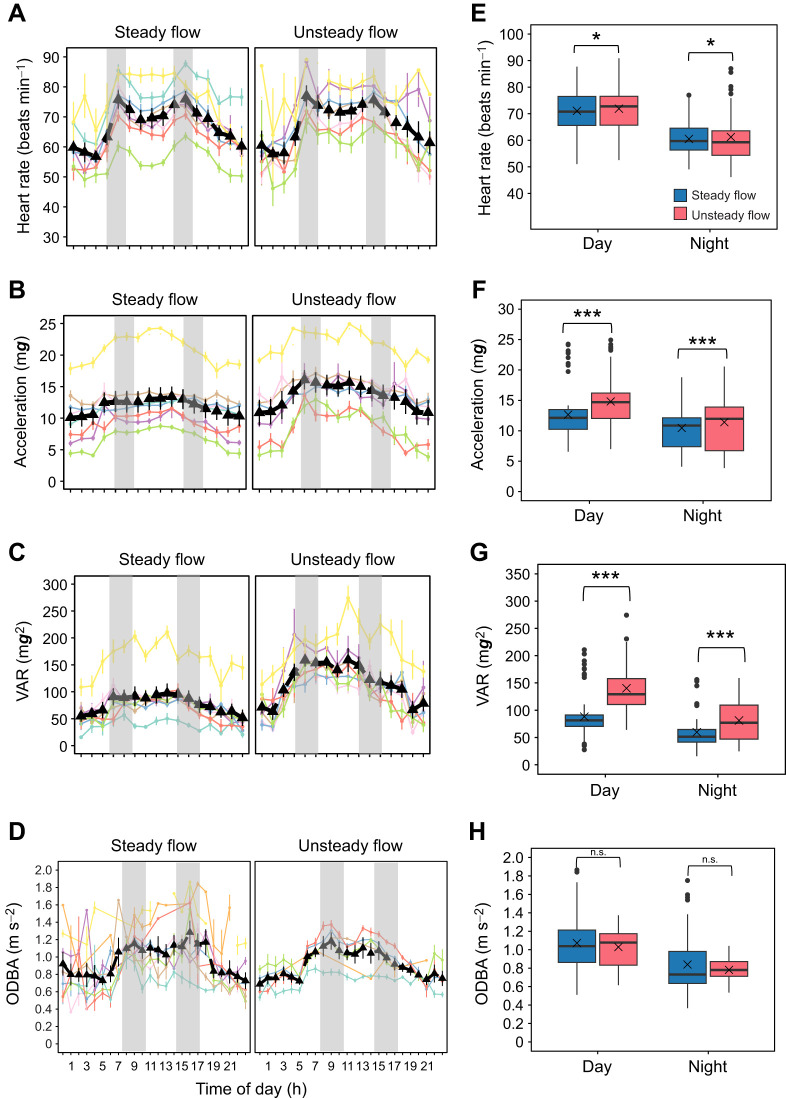
**Hourly and diel variations in heart rate (HR) and acceleration dynamics of post-smolt Chinook salmon under steady and unsteady flow.** (A–D) Hourly HR, acceleration, acceleration variance, and overall dynamic body acceleration (ODBA); individual values are shown as coloured lines, means as triangles ±s.e.m, and shaded areas indicate feeding times. (E–H) Day (07:00–17:00 h) versus night (18:00–05:00 h) box plots for the same variables. Blue and red denote steady and unsteady flow, respectively. Asterisks indicate significant differences (*P*<0.05; LMM); n.s. denotes non-significance.

Hourly patterns of HR, acceleration, VAR and ODBA in co-implanted fish swimming freely at 0.50 m s^−1^ under steady or unsteady flow (0.50±0.05 m s^−1^) are shown in Fig. 7. Regardless of flow condition, all metrics except ODBA were significantly higher during the day than at night (LMM, *P*<0.05). Over the 4 week trial, fish body mass increased by 27% (initial mean: 426 g) and total length by 9% (initial mean: 32 cm). Two mortalities were recorded among non-implanted fish.

Mean HR of fish was significantly higher under unsteady flow conditions than under steady flow, both during the day (73.20±2.53 versus 71.10±2.52 beats min^−1^) and at night (62.50±2.55 versus 60.50±2.54 beats min^−1^; LMM, *P*=0.02). Although the mean HR difference between flow conditions was small (∼2 beats min^−1^), the effect was statistically significant owing to the consistency of repeated HR measurements across fish and time, as captured by the mixed-effects structure. Notably, the magnitude of the circadian HR fluctuation (∼11 beats min^−1^) remained consistent across both flow regimes (Fig. 7A,E). Acceleration also differed significantly between flow conditions (LMM, *P*<0.001; conditional *R*^2^=0.90). During the day, acceleration was higher under unsteady flow (14.60±1.48 m***g***) than under steady flow (12.90±1.47 m***g***) conditions, and similarly at night (11.80±1.48 versus 10.10±1.48 m***g***; Fig. 7B,F). VAR was significantly greater under unsteady flow conditions both during the day (128±12.3 m***g***^2^) and night (86±12.4 m***g***^2^) compared with steady flow conditions (90±12.20 m***g***^2^ day; 51±12.31 m***g***^2^ night; LMM, *P*<0.001; Fig. 7C,G). Although daytime ODBA values were higher than night-time values under both flow conditions, values between steady and unsteady flow were statistically similar (day: 1.13±0.07 versus 1.10±0.07 m s^−2^; night: 0.88±0.07 versus 0.86±0.07 m s^−2^; LMM, *P*=0.34; Fig. 7D,H).

## DISCUSSION

In this study, the heart rate and activity dynamics of Chinook salmon were investigated during a swim-exercise test in a swim tunnel. This test was carried out under both steady and unsteady flow conditions while *Ṁ*_O_2__ was determined. HR, acceleration and ODBA values of co-implanted fish were compared with fish that were implanted with either a logger or transmitter, and with sham and control groups to ensure tag implantation did not affect variables measured. Insights were obtained not only from HR and acceleration measurements in fish in swim tunnel experiments, but also in free-swimming fish under steady and unsteady flow conditions in large tanks. The relationship between acceleration and ODBA values as recorded in co-implanted fish during the swim tunnel experiment was investigated. Regression models were developed for acceleration, ODBA and *Ṁ*_O_2__ versus swimming speed. We hypothesized that swimming under unsteady flow conditions would be energetically more costly (higher *Ṁ*_O_2__) for Chinook salmon than when swimming under steady flow conditions, thus similar as observed for Atlantic salmon (Agbeti et al., 2024). Furthermore, we hypothesized that HR, acceleration and ODBA patterns of Chinook salmon would be higher under unsteady flow compared with steady flow conditions in free-swimming fish.

HR was unaffected by swimming at increasing speeds under either steady or unsteady flow conditions in the swim tunnel. HR values were already high (82 beats min^−1^) at the lowest speed of 0.15 m s^−1^ and showed no substantial increase with swimming speeds from 0.3 to 0.9 m s^−1^ (Fig. 4A), despite substantial increases of *Ṁ*_O_2__ (Fig. 3A), acceleration (Fig. 4B) and ODBA (Fig. 5A). Consequently, no correlation was observed between HR and *Ṁ*_O_2__, HR and acceleration, nor HR and ODBA. This apparent decoupling of cardiac and metabolic responses suggests that Chinook salmon maintained aerobic performance primarily through adjustments in stroke volume or peripheral oxygen extraction, rather than through HR. Such compensation has been documented in salmonids that can increase stroke volume threefold during strenuous exercise (Eliason et al., 2013; Farrell et al., 2009; Thorarensen et al., 1996), thereby maintaining oxygen delivery when HR scope is limited. The apparent maximal HR of Chinook salmon at the start of the swim tunnel trial may still reflect stress from netting, anaesthesia, individual housing in the swim tunnel, further compounded by their skittish behaviour, as documented elsewhere (Elvy et al., 2022; Prescott et al., 2023). These factors may therefore mask any exercise-induced HR increase, thereby limiting dynamic range during swimming. Nevertheless, 75% of the co-implanted fish did not fatigue during trials, whereas the remaining 25% fatigued at 0.8 m s^−1^ under the steady flow condition. The ability of Chinook salmon to sustain aerobic performance at near-maximal HR suggests efficient cardiovascular regulation that supports oxygen delivery under variable hydrodynamic conditions. The capacity to meet these demands, possibly by adjusting stroke volume and oxygen extraction may therefore represent an adaptive strategy for coping with variable flow environments. Future research should investigate cardiac stroke volume, arterial–venous oxygen difference and autonomic regulation under unsteady flow to elucidate how Chinook salmon balance cardiovascular efficiency and environmental variability.

The acceleration of Chinook salmon, in contrast, showed a linear increase with swimming speed but only within the range of 0.30 to 0.75 m s^−1^ and regardless of the flow condition (Fig. 4B). When swimming at these speeds, the mean acceleration of fish nearly doubled (1.89-fold). This result reflects the progressive recruitment of muscle fibres along with increased tail-beat amplitude and frequency as swimming speed increases. However, at 0.90 m s^−1^, mean acceleration value dropped from 17.5±1.29 to 14.30±0.88 m***g*** and from 17.5±1.49 to 15.31±1.24 m***g*** for fish swimming under steady and unsteady flow conditions, respectively (Fig. 4B). The drop in acceleration values of Chinook salmon at 0.90 m s^−1^ suggests transition from sustained aerobic swimming to burst and glide swimming behaviour. This reduction probably indicates depletion of muscle energy reserves and reduced thrust production as fish approached their threshold, where anaerobic white muscle is recruited transiently to maintain forward momentum (Webb, 1975). Based on the *Ṁ*_O_2__ profiles, the onset of burst-and-glide swimming appeared to occur at approximately 0.75 m s^−1^, indicating that this speed represents the transition point where continuous aerobic swimming could no longer be maintained. In our previous research with Atlantic salmon of similar size (∼471 g), implanted with an HR/acceleration data logger and swimming under steady flow conditions, we observed a comparable twofold increase in acceleration values (Agbeti et al., 2025). In that study, the increase in acceleration occurred when fish were swimming between 0.2 and 1.0 m s^−1^, with speed increments of 0.2 m s^−1^ every 60 min until fatigue. Similarly, Zrini and Gamperl (2021) reported a 2.5-fold increase in acceleration values of ∼740 g Atlantic salmon during an exercise test ranging between 0.3 and 1.0 m s^−1^. Greater factorial increases in acceleration values with swimming speed, up to sixfold, have been observed in other non-salmonid species, such as the European sea bass (*Dicentrarchus labrax*) (Wright et al., 2014). To better understand the general relationship between acceleration and *Ṁ*_O_2__ of fish, we plotted the mean values of the two parameters. Mean acceleration values increased linearly with mean *Ṁ*_O_2__ under both steady (*R*^2^=0.99) and unsteady (*R*^2^=0.95) flow conditions (Fig. 4C), consistent with the tight coupling between mechanical effort and metabolic rate observed in other salmonids (Agbeti et al., 2025; Clark et al., 2010). This relationship underscores that acceleration integrates both the frequency and amplitude of swimming movements, effectively serving as a biomechanical proxy for aerobic energy expenditure. Likewise, ODBA of Chinook salmon increased with swimming speed (Fig. 5A), and mean ODBA was positively and linearly correlated with *Ṁ*_O_2__ under both steady (*R*^2^=0.69) and unsteady (*R*^2^=0.71) flow conditions (Fig. 5B). This is consistent with patterns in other species where ODBA scales with mechanical power output (Agbeti et al., 2024; Wilson et al., 2013). Also, mean ODBA and acceleration values, obtained from sensors that were co-implanted in the experimental fish swimming at increasing speed, co-varied substantially in a positively linear manner (Fig. 6). This relation was found under both steady and unsteady flow conditions. The positive co-variation between ODBA and acceleration (Fig. 6) reinforces that both measures capture the magnitude of body motion and swimming effort, reflecting underlying muscle activity and mechanical work (Qasem et al., 2012). From a functional perspective, these results suggest that Chinook salmon modulate swimming activity proportionally to metabolic demand across a wide range of flow conditions. The consistent acceleration–*Ṁ*_O_2__ relationship under unsteady flow implies that Chinook maintain efficient kinematic control even in energetically variable environments, an ability that is likely to be advantageous in open ocean aquaculture sites characterized by unsteady flow conditions and fluctuating currents. Thus, beyond their applied relevance for sensor-based monitoring, acceleration and ODBA metrics provide fundamental insight into the biomechanical and physiological coupling of locomotion and metabolism in fish adapted to unsteady conditions.

Chinook salmon swimming in unsteady flow displayed marginally higher *Ṁ*_O_2__ (5–15%) with increasing swimming speeds compared with when swimming under steady flow condition. Although this difference was not statistically significant, the data suggested a weak trend. This result was contrary to our hypothesis and contrasts with the stronger metabolic response reported for Atlantic salmon (15–53%; Agbeti et al., 2024) and shiner perch (*Cymatogaster aggregata*) (25%; Roche et al., 2014). One plausible explanation for the absence of a significant difference in Chinook salmon may relate to the shorter recovery period (∼2 h) following handling, anaesthesia and introduction into the swim tunnel prior to testing. Residual handling stress probably elevated baseline metabolic rates at the start of the trials, thereby reducing the scope for further increases in *Ṁ*_O_2__ during swimming. In addition to this experimental consideration, species-specific factors may have also contributed to the weak *Ṁ*_O_2__ response. Chinook salmon possess a more fusiform body shape and greater reliance on continuous, steady propulsion powered by red muscle fibres (Petrell and Jones, 2000). This may allow them to maintain efficient thrust production even in unsteady flow. Their ecological background as strong upstream migrants (Kraskura et al., 2024) also suggests an inherent tolerance for fluctuating velocity fields, enabling energy conservation through phase-synchronized tail beats when encountering oscillating currents. Nevertheless, the consistent increase in *Ṁ*_O_2__ with swimming speed under both flow conditions supports the expected relationship between metabolic rate and exercise intensity, as previously observed in Chinook salmon (Prescott et al., 2023) and other salmonids (Agbeti et al., 2024; Eliason et al., 2013; Hvas et al., 2021a; Remen et al., 2016).

Regarding free-swimming Chinook salmon in tanks, HR variations of co-implanted fish followed a circadian pattern, displaying an M-shaped trend, with higher HR values during the day, peaking at morning and afternoon feedings (Fig. 7). Estimated daytime HR values were 71 and 73 beats min^−1^ under steady and unsteady flow conditions, respectively, approximately 11 beats min^−1^ higher than night-time HR values (Fig. 7A,E). The circadian HR pattern observed in free-swimming fish was consistent with changes in activity, as indicated by acceleration, VAR and ODBA trends (Fig. 7B,C,D,F,G,H), reflecting coordinated regulation of cardiovascular and locomotor systems. The stability of circadian rhythms in our Chinook salmon suggests adequate recovery and acclimation following surgery and tank reintegration, allowing endogenous physiological cycles to re-emerge. Similar circadian HR patterns have been reported in rainbow trout (*Oncorhynchus mykiss*) under aquaculture conditions, although patterns were noticeable only after 4 days of recovery (Brijs et al., 2018). In that study, initial disruptions were attributed to stress from surgery, transport and reintegration with conspecifics in sea cages. In non-salmonid species such as gilthead seabream (*Sparus aurata*), acceleration values, as recorded by implantable acoustic transmitter tag, showed a W-shaped pattern when monitored continuously for 6 weeks in sea cages (Palstra et al., 2021). The authors reported daily rhythms in swimming activity under the experimental conditions characterized by more active periods at 06:00–14:00 h and 18:00–24:00 h and less active periods at 00:00–06:00 h and 14:00–18:00 h. In our study, we found that VAR values were higher under unsteady flow conditions than under steady flow conditions. A VAR value above 222 m***g***^2^ in Atlantic salmon likely indicates burst–coast swimming or distress behaviours (Zrini and Gamperl, 2021). In our study, mean VAR values of free-swimming salmon under steady flow remained below 100 m***g***^2^. This implies efficient movement, where fish maintain a steady pace, reducing energy expenditure. The substantially higher VAR under unsteady flow conditions suggests adapting to the dynamic conditions which could result in high energy use. Additionally, one fish exhibited an exceptionally high daytime VAR, regardless of flow conditions, suggesting potential erratic swimming behaviour due to distress (Fig. 7C; yellow data trend). When consistently observed with sensor implants, such erratic behaviour could be a useful indicator for predicting the behavioural and possibly physiological status of an individual fish or a cohort.

Based on the swimming activity and oxygen use of Chinook salmon in the swim tunnel, we attempted to predict acceleration, ODBA and *Ṁ*_O_2__ in the free-swimming fish. From acceleration versus swimming speed and ODBA versus swimming speed regression equations, as derived from the respirometry studies on Chinook salmon (Figs 4B and 5A), we predicted that fish swimming at 0.50 m s^−1^ under steady flow conditions would exhibit acceleration values of 13.04 m***g*** and ODBA values of 1.06 m s^−2^ respectively. Under unsteady flow conditions, fish were predicted to show acceleration and ODBA values of 13.74 m***g*** and 1.33 m s^−2^ respectively. Observed values, obtained from free-swimming fish, closely aligned with the predicted values. Under steady flow, mean acceleration and ODBA values found were 12.90 m***g*** and 1.12 m s^−2^, respectively. Under unsteady flow, these values were 14.60 m***g*** and 1.09 m s^−2^, respectively. The similarity between predicted acceleration and ODBA values obtained from respirometry-based regression equations with these observed values in free-swimming fish suggests the possibility of accurate estimation of metabolic rates using these parameters. Using acceleration/*Ṁ*_O_2__ (Fig. 4C) and ODBA/*Ṁ*_O_2__ (Fig. 5B) regression models from the respirometry study, estimated *Ṁ*_O_2__ values for free-swimming fish in the experimental tanks were 240 and 241 mg kg^−1^ h^−1^ under steady flow conditions respectively, and 247 and 253 mg kg^−1^ h^−1^ under unsteady flow conditions respectively. This demonstrates the potential of using sensor implants to assess activity-related energy expenditure in cultured Chinook salmon and potentially in wild populations.

## Conclusion

HR in Chinook salmon reached apparent maximum when introduced in the swim tunnels, which did not increase further at increasing swimming speeds under steady or unsteady flow conditions. In contrast, both acceleration and ODBA increased when higher swimming speeds were applied under both flow conditions. In free-swimming fish, HR, acceleration, VAR and ODBA followed a circadian pattern, with higher mean values under unsteady flow compared with steady flow conditions, except for ODBA, which was comparable under both conditions. Acceleration and ODBA were positively and linearly correlated with swimming speed, particularly when swimming in the swim tunnels between speeds of 0.30 and 0.75 m s^−1^. Regression models of acceleration and ODBA relative to swimming speed closely predicted the values observed in free-swimming fish. Findings indicate how swimming speeds and *Ṁ*_O_2__, HR, acceleration and ODBA inter-relate under steady and unsteady flow conditions, which could be used for characterizing the swimming performance of fish in offshore farming environments.

## Supplementary Material

10.1242/jexbio.251222_sup1 Supplementary information
