# Supplementary material for: Heart rate and activity patterns of chinook salmon (Oncorhynchus tshawytscha) under steady and unsteady flow conditions
Source: J Exp Biol. 2026 Mar 30;229(7):jeb251222. doi: 10.1242/jeb.251222 (PMC13086490; doi:10.1242/jeb.251222)
Supplement: Supplementary information [file jexbio-229-251222-s1.pdf]

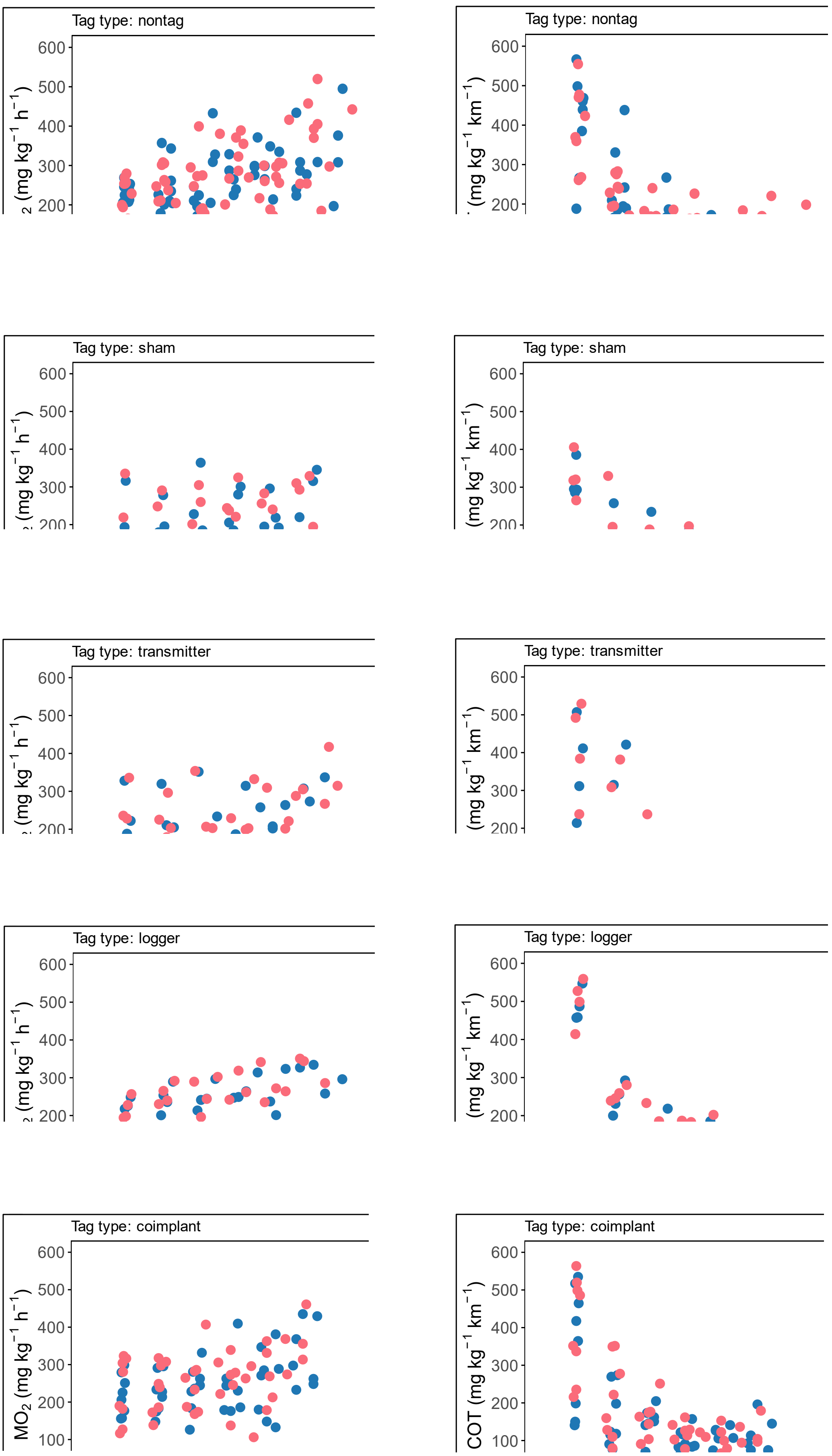

Journal of Experimental Biology • Supplementary information

Fig S1. Oxygen consumption (MO_2) and Cost of Transport (COT) of different fish groups across swimming speeds under both steady (blue) and unsteady (red) flow conditions. Panels (A–E) show MO2 vs. swimming speed (plotted as body length per second; bl s^{-1}) for control (n=12), sham (n=4), transmitter (n=4), logger (n=4), and co-implanted (n=8) groups, respectively. Panels (F–J) present corresponding COT values for the same groups. No significant differences in MO_2 or COT were observed between groups or flow conditions (LMM, $P>0.05$).

Table S1. Biometric parameters of experimental post-smolt Chinook salmon (*Oncorhynchus tshawytscha*) after a swim test in a swim tunnel. Body weight (BW), standard length (SL) and condition factor (K) are presented for fish in the control, sham, transmitter, logger, and co-implant groups, swimming under both steady and unsteady flow conditions. Number of fish is represented by n. Comparisons were made among fish groups within each flow condition using the Kruskal-Wallis test. Comparisons within the similar fish group between steady and unsteady flow conditions were performed using the Wilcoxon signed-rank test. Groups showed no significant differences ($P>0.05$) within and between flow conditions. Values are given as mean±s.d.

	**Control**	**sham**	**Transmitter**	**Logger**	**Co-implants**
**Steady flow**					
**n**	12	4	4	4	8
**BW (g)**	432±104	552±46	426±72	462±103	562±111
**SL (cm)**	29.83±2.42	31.63±1.11	29.88±2.14	30±1.96	31.88±1.36
**K**	1.60±0.09	1.65±0.08	1.59±0.05	1.69±0.05	1.72±0.05
**Unsteady flow**					
**n**	12	4	4	4	8
**BW (g)**	465±120	521±58	417±86	466±92	561±90
**SL (cm)**	30.38±2.44	31.75±0.87	29.75±1.94	30.13±2.53	32.13±1.90
**K**	1.63±0.04	1.56±0.13	1.57±0.01	1.70±0.08	1.68±0.03

Table S2. Comparison of optimal swimming speed of post-smolt Chinook salmon (*Oncorhynchus tshawytscha*). Results are presented for fish in each group: control, sham, transmitter, logger, and co-implant, under both steady and unsteady flow conditions. Comparison was made among fish groups within each flow condition using the Kruskal-Wallis test. Comparisons between fish groups under steady and unsteady flow conditions were performed using the Wilcoxon signed-rank test. Groups showed no significant differences (P>0.05) within and between flow conditions. The maximum swimming speed tested in this experiment was 1.0 m s^{-1}.

	Control	sham	Transmitter	Logger	Co-implants
Steady flow					
n	12	4	4	4	8
U_{opt} (m s^{-1})	0.56±0.03	0.52±0.06	0.62±0.02	0.61±0.03	0.59±0.03
Unsteady flow					
n	12	4	4	4	8
U_{opt} (m s^{-1})	0.57±0.02	0.46±0.05	0.61±0.03	0.66±0.04	0.60±0.03
